# Multimodal intelligent logistics robot combining 3D CNN, LSTM, and visual SLAM for path planning and control

**DOI:** 10.3389/fnbot.2023.1285673

**Published:** 2023-10-16

**Authors:** Zhuqin Han

**Affiliations:** School of Intelligent Engineering, Shaoguan University, Shaoguan, China

**Keywords:** multimodal logistics robot, 3D CNN, LSTM, Dijkstra, SLAM, path planning

## Abstract

**Introduction:**

In today's dynamic logistics landscape, the role of intelligent robots is paramount for enhancing efficiency, reducing costs, and ensuring safety. Traditional path planning methods often struggle to adapt to changing environments, resulting in issues like collisions and conflicts. This research addresses the challenge of path planning and control for logistics robots operating in complex environments. The proposed method aims to integrate information from various perception sources to enhance path planning and obstacle avoidance, thereby increasing the autonomy and reliability of logistics robots.

**Methods:**

The method presented in this paper begins by employing a 3D Convolutional Neural Network (CNN) to learn feature representations of objects within the environment, enabling object recognition. Subsequently, Long Short-Term Memory (LSTM) models are utilized to capture spatio-temporal features and predict the behavior and trajectories of dynamic obstacles. This predictive capability empowers robots to more accurately anticipate the future positions of obstacles in intricate settings, thereby mitigating potential collision risks. Finally, the Dijkstra algorithm is employed for path planning and control decisions to ensure the selection of optimal paths across diverse scenarios.

**Results:**

In a series of rigorous experiments, the proposed method outperforms traditional approaches in terms of both path planning accuracy and obstacle avoidance performance. These substantial improvements underscore the efficacy of the intelligent path planning and control scheme.

**Discussion:**

This research contributes to enhancing the practicality of logistics robots in complex environments, thereby fostering increased efficiency and safety within the logistics industry. By combining object recognition, spatio-temporal modeling, and optimized path planning, the proposed method enables logistics robots to navigate intricate scenarios with higher precision and reliability, ultimately advancing the capabilities of autonomous logistics operations.

## 1. Introduction

In today's context of globalization and digitization, the logistics industry is facing increasing challenges. Improving logistics efficiency, reducing costs, and ensuring the safety and accuracy of transportation has become an urgent need (Duan, 2018; Xue et al., 2021). The development of intelligent robotics offers new possibilities to address these challenges. In recent years, deep learning technology has achieved great success in the fields of computer vision and natural language processing, a multimodal deep learning framework proposed in Yao et al. (2023) and some novel networks (Wu et al., 2022; Li et al., 2023) providing new ideas and methods for path planning and control of intelligent logistics robots (Bernardo et al., 2022). Below are some commonly used deep learning methods for path planning of logistics robots.

Convolutional Neural Network (CNN) (Wu et al., 2021): CNN is a neural network that can be used for visual perception, capturing scenes through cameras, detecting obstacles, signs, and landmarks, thereby creating real-time environmental maps. The robot also uses CNN to analyze sensor data in real time to avoid obstacles while in motion. This helps the robot better understand its surroundings and thus navigate more safely.

Recurrent Neural Network (RNN) (Li et al., 2018): Intelligent logistics robots usually need to process time-related data, such as sensor data, map information, and previous movement history. RNN is widely used to model these time-series data, which can capture the time-series dependencies in the data and help robots better understand and predict changes in the environment. Among other things, RNN are used for path planning, especially when historical information needs to be considered and predictions of future states are required. By modeling previous paths, motion history, and environmental changes, RNN can help robots predict optimal paths to avoid obstacles or adapt to different work scenarios.

Support Vector Machine (SVM) (Tong et al., 2019): SVM is widely used in environment perception and obstacle recognition. By treating different types of landmarks, objects or obstacles as different categories, SVM can help robots categorize their environment to better understand the world around them. This helps smart logistics robots make smarter decisions. And SVM supports online learning, which means that the robot can continuously adjust its path planning and control strategy based on the data collected in real time. This ability enables robots to adapt to changing environments, dealing with unknown obstacles and new tasks.

Reinforcement Learning (RL) (Choi et al., 2021): RL allows robots to learn from their interactions with the environment to optimize path planning and decision making. Robots learn optimal paths and action strategies by interacting with the environment, for example, simulating movement and avoiding obstacles to achieve task optimality or achievement of a specific goal.

Deep Reinforcement Learning (DRL) (Yang et al., 2020): DRL can help robots optimize resource allocation, such as minimizing time and energy or cost, to improve efficiency and economy. DRL allows robots to learn directly from sensor data, enabling end-to-end learning and decision making. This eliminates the need for manual feature engineering in traditional approaches and simplifies system design.

There are other commonly used models including but not limited to the above five models:

1. The Generative Recurrent Neural Networks (GRNN):

Generative Recursive Neural Networks (GRNN) (Ren et al., 2022) is a neural network model for generating tree-structured data. Its core idea is to recursively decompose the tree structure into subtrees, and then combine the representations of the subtrees into parent nodes. To generate a representation of the entire tree. GRNN recursively processes the structure of the tree, splits the tree into subtrees, and then combines the representations of the subtrees into the representation of the parent nodes. This process is similar to the phrase structure analysis in natural language. By continuously recursively processing nodes and edges, a representation of the entire tree is finally generated. For each node, GRNN will learn a representation vector to capture the semantic information of the node. This can be achieved by using embedding layers or other neural network layers such that each node has a fixed-dimensional vector representation. For each parent node, GRNN combines the representations of its child nodes to generate a representation of the parent node. This usually involves combining the representation vectors of the child nodes, for example using RNNs, LSTMs. By continuously recursively combining child node representations, the representation of the root node of the entire tree is finally generated (Dudukcu et al., 2023).

GRNN model has a wide range of applications in the path planning and control of multi-modal intelligent logistics robots (Ma et al., 2021). GRNN is an extension of Recurrent Neural Network, which focuses on generating sequence data, such as time series, language sequences. In path planning and control, GRNN can be used to predict object movement, behavior patterns, and environmental changes, thereby achieving more accurate path planning and obstacle avoidance decisions. GRNNs are designed for sequence data and can capture temporal changes and patterns of objects and obstacles. This is very helpful in predicting the behavior and trajectory of dynamic obstacles, allowing better path planning for the robot. The generative performance of GRNNs enables them to predict future sequence data, such as the future positions of dynamic obstacles. This ability is useful in path planning, helping the robot avoid collisions with possible future obstacles. GRNN can adapt to many types of input data, including images, sounds. This makes it flexible in path planning and control in multimodal intelligent logistics robots, which can acquire information from different perception modalities. The disadvantage is that GRNN requires a large amount of time series data for training. Similar to traditional RNNs, GRNNs may also face long-term dependency problems when processing long sequences. This can lead to inaccurate forecasts on longer time horizons. Moreover, the GRNN model is relatively complex, and the computational cost of training and inference may be high. In real-time applications, the use of computing resources needs to be considered.

2. Deep Hierarchical Reinforcement Learning:

Deep Hierarchical Reinforcement Learning (DHRL) (Zhao et al., 2019) is a method to introduce hierarchical structure in reinforcement learning, aiming at solving exploration and policy learning problems in complex tasks. DHRL reduces the difficulty of exploration and improves learning efficiency by decomposing the task into multiple subtasks, each with its own policy.

In DHRL, tasks are broken down into multiple levels of subtasks. These subtasks can be designed according to the complexity and difficulty of the task, so that each subtask can be solved more simply (Lee G. et al., 2022). DHRL introduces two levels of policies, namely the master policy (meta-policy) and the slave policy (lower-level policy). The master strategy decides when to switch subtasks, while the slave strategy is responsible for executing the current subtask. Each subtask has its own reward function, which is used to evaluate the performance of the slave policy. These reward functions can be designed according to the goals and requirements of the subtasks to guide the learning from the policy. By alternately training master and slave policies, the DHRL system learns how to select subtasks and execute them. The main strategy decides which subtask to switch to based on the current state and the performance of the subtasks. According to the current subtask and status, the slave strategy selects an action to execute. Through continuous learning and optimization, the DHRL system gradually adjusts the subtask strategy and the decision-making process of the main strategy. The learning of the master policy can be based on the performance and reward function of the subtasks, and the learning of the slave policy can use traditional reinforcement learning methods.

Deep Hierarchical Reinforcement Learning has potential applications in the field of path planning and control of multi-modal intelligent logistics robots. The method enables higher-level decision-making and path planning by combining reinforcement learning with hierarchical structures, thereby improving robot performance in complex environments (Lee S. et al., 2022). The advantage is that Deep Hierarchical RL allows robots to make decisions at different levels, from high-level task planning to low-level motion control. This hierarchical decision-making allows the robot to respond more efficiently to different scenarios and tasks. Through hierarchies, robots are able to learn high-level abstract representations to understand larger-scale features of the environment. This facilitates more accurate path planning and decision-making, while improving adaptability to complex environments. Hierarchical structure can share and reuse knowledge among different tasks. This means that after the robot has learned one task, it can apply what it has learned to other tasks, speeding up the learning process. The downside is that the hierarchical structure makes the algorithm more complex and requires careful design and tuning. The complexity of the algorithm can make the training and inference process more time-consuming. The design of the hierarchical structure requires a deep understanding of the problem domain and tasks. Improper layered design can lead to performance degradation. Hierarchical reinforcement learning requires training at multiple levels, potentially requiring more samples and training time, especially in complex environments.

3. Simultaneous Localization and Mapping (SLAM):

Simultaneous Localization and Mapping (SLAM) (Pak et al., 2022) is an integrated technology for simultaneous positioning and map construction of robots in unknown environments. It is a key technology to realize robot position estimation and environment map establishment through sensor data without prior map. SLAM has a wide range of applications in the field of robotics, including autonomous driving, drones, mobile robots. The SLAM system collects environmental information through sensors (such as lidar, camera, inertial measurement unit.), and these data are usually unstructured and noisy. In SLAM, the robot needs to estimate its own position, that is, determine its own coordinates on the map. This process is called localization and can be achieved based on sensor data and previous position estimates. The accuracy of localization is crucial to the whole SLAM process. At the same time, the SLAM system needs to build a map of the environment, that is, map the perception data to the location in the real world. Maps can be 2D or 3D and include information such as obstacles, feature points, and landmarks. In SLAM, state estimation and optimization are required, i.e., computing the most probable robot position and map based on perception data and previous estimates. This usually involves using a probabilistic filter (e.g., extended Kalman filter, particle filter) to maintain the estimated state. SLAM systems also need to deal with loop closure, that is, passing through the same locations while exploring the environment. Closed loops may lead to deviations in position estimation, so it is necessary to correct the estimation error by detecting closed loops and making adjustments to the previous state. SLAM realizes autonomous positioning and map construction of robots in unknown environments by fusing sensor data, thereby providing key information for path planning and obstacle avoidance (Li et al., 2019). The advantage is that SLAM can help the robot perceive and understand its environment, including map information, obstacle locations. This perception capability is critical for path planning and obstacle avoidance decisions. SLAM can achieve precise positioning of the robot, thereby providing accurate starting points and reference information for path planning. This is crucial for efficient path planning. SLAM can automatically generate maps during robot exploration, which provides valuable background information for path planning, enabling robots to better understand the environment and make decisions. The downside is that SLAM is computationally expensive, especially in real-time applications. For path planning and control tasks that require fast response, algorithms may need to be optimized or computing resources increased (Alsadik and Karam, 2021). SLAM requires a variety of sensor data, such as vision, lidar, to fuse different information to achieve environmental perception. However, high-quality sensors can be expensive, and low-quality sensors can affect the accuracy of SLAM. SLAM can be challenging in dealing with dynamic environments, such as moving obstacles that can interfere with the localization and mapping process, thereby affecting path planning and obstacle avoidance.

4. Path Planning and Obstacle Avoidance in the Field of Intelligent Robotics:

Path planning and obstacle avoidance are issues of significant concern in the field of intelligent robotics, particularly in complex environments. The research by Cai et al. offers a unique perspective on robot development by closely linking robots with their living spaces. This literature emphasizes the importance of robots perceiving and adapting to their surroundings, a viewpoint closely aligned with the theme of this study. In the context of path planning and obstacle avoidance, understanding how robots interact with their living spaces and perceive the surrounding environment is paramount to addressing challenges within complex environments (Cai et al., 2021). Jiao et al.'s research introduces a method that combines convolutional neural networks (CNNs) and bidirectional long short-term memory networks (bi-LSTMs) for monocular visual odometry. Although their specific techniques differ from the 3D CNN and LSTM used in this study, this work demonstrates the potential of deep learning approaches in handling perceptual data. In intelligent robot path planning, the accuracy and understanding of perceptual data are of paramount importance, making this reference valuable for insights into perception technology (Jiao et al., 2019). Wenxi et al.'s comprehensive survey investigates the field of social computing involving the collaboration between artificial intelligence and humans. While seemingly unrelated to logistics robot path planning, this literature offers insights into multimodal information fusion and intelligent system decision-making. In this study, we integrate various perceptual modalities, including images, point clouds, and dynamic obstacle trajectories, to improve path planning and obstacle avoidance control. The perspective presented in this reference inspires our consideration of the importance of multimodal information fusion (Wang et al., 2022).

In summary, these related works provide essential background information for this study on path planning, perception technologies, and the interaction of robots with their environment. They highlight the complexity of path planning and obstacle avoidance problems in complex environments and offer some methods and perspectives to address these challenges. In this research, we integrate insights from these works and adopt a comprehensive approach to enhance the performance and reliability of intelligent logistics robots.

Intelligent logistics robots have great potential in achieving efficient, safe, and precise path planning and control, however, the limitations of traditional methods in complex environments prompt us to seek innovative solutions. In this paper, 3D CNN is used for feature extraction, LSTM is used for timing modeling, and visual SLAM technology is used to realize the positioning and mapping of robots in unknown environments. Finally, the Dijkstra algorithm is used for path planning.

This paper combines 3D CNN, LSTM, visual SLAM, and Dijkstra's algorithm to achieve efficient path planning and obstacle avoidance control for intelligent logistics. Firstly, in complex logistics environments, intelligent logistics robots require a high level of perception and planning capabilities to cope with constantly changing obstacles and environmental conditions. Traditional path planning methods may not offer sufficient performance and reliability in these complex scenarios. Therefore, a comprehensive approach that integrates multiple advanced technologies can better address these challenges. Secondly, different perception modalities provide rich information, including images, point clouds, and trajectories of dynamic obstacles. Leveraging this information can enhance the accuracy of path planning and obstacle avoidance. For instance, 3D CNN is used for object recognition, LSTM for predicting the behavior of dynamic obstacles, visual SLAM for map construction, and Dijkstra's algorithm for path planning. These technologies work in synergy to enable the robot to better understand its surroundings and make appropriate planning decisions. Furthermore, a single model may have limitations, whereas combining multiple technologies can compensate for each other's shortcomings. For example, the traditional Dijkstra's algorithm can provide the optimal path but may not consider the impact of dynamic obstacles. 3D CNN and LSTM are better suited to handle this dynamism. The objective of the integrated model is to harness the strengths of various technologies to provide more efficient and safer path planning and obstacle avoidance performance in complex environments.

Through the above methods, this paper aims to break the limitations of traditional path planning and control methods, and provide more efficient and safe path planning and control capabilities for intelligent logistics robots in complex and changeable environments.

The contribution points of this paper are as follows:

- Innovative application of multi-modal intelligent path planning method: This paper organically combines multi-modal perception and deep learning technologies such as 3D CNN, LSTM and visual SLAM for the first time, providing an innovative solution for intelligent logistics robot path planning and control. By extracting spatio-temporal features of image sequences and modeling dynamic obstacles, robots can more accurately predict the position and motion of objects in the environment, thereby avoiding collisions in path planning and improving the accuracy and reliability of path planning.
- Intelligent improvement of path planning decision-making: Combining LSTM modeling and Dijkstra algorithm, the method proposed in this paper enables robots to adaptively plan paths in dynamic environments. By predicting the future behavior of obstacles, robots can make more sensible path choices in complex scenarios, effectively avoid collisions and conflicts, and improve the safety and efficiency of logistics systems.
- Improve logistics efficiency and reduce costs: The research in this paper can help intelligent logistics robots achieve precise path planning and control. This will significantly improve the efficiency of logistics operations, reduce waste of time and energy, reduce logistics costs and improve the competitiveness of the overall logistics system.

The logical structure of this article is as follows: In Section 2, the methods section, this article elaborates on the technical roadmap of the proposed method and the inference process of each module in detail. In Section 3, the experimental section, the article describes information such as the experimental environment configuration and dataset sources. It introduces the evaluation metrics used and provides numerous tables and graphics to showcase the performance comparison results of different methods. Through abundant experimental data, this article comprehensively validates the effectiveness of the proposed method. In Section 4, the conclusion and discussion section, the research work is summarized, the research significance is analyzed, limitations are discussed, and future research directions are outlined.

## 2. Methodology

### 2.1. Overview of our network

**Figure 1:**
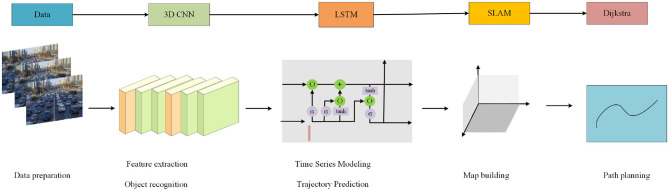
Overall flow chart of the model.

This method aims to combine a variety of advanced technologies, 3D CNN, LSTM, visual SLAM and Dijkstra algorithm, to achieve efficient path planning and obstacle avoidance control for intelligent logistics robots. The method covers key steps such as object recognition, obstacle prediction, and path planning to improve the autonomy and safety of logistics robots. Figure 1 is the overall flow chart:

The overall implementation process of the method in this paper is: data processing, feature extraction and object recognition, time series modeling and trajectory prediction of dynamic obstacles, visual SLAM positioning and map construction, path planning and decision-making. First, randomly select the data in the data set for processing; Second, 3D CNN is used to extract spatio-temporal features from image sequences in the environment to realize object recognition and obstacle detection. The model learns feature representations of different objects in the environment to accurately identify obstacles in real-time images. Then, the feature sequence extracted by 3D CNN is input into LSTM to model the trajectory and behavior pattern of dynamic obstacles. Because LSTM can predict the future position of obstacles by learning historical data, so as to avoid the risk of collision in path planning. Then, combined with visual SLAM technology, localization and map construction are realized in unknown environments. Finally, use Dijkstra's algorithm for path planning. Algorithms combine obstacle position and motion information to ensure that the robot chooses the optimal path to avoid collisions with obstacles. Through the above process, the method can realize the path planning and control of the intelligent logistics robot, improve the logistics efficiency, reduce the risk, and also provide a useful reference for technological innovation in the field of intelligent robots.

### 2.2. 3D convolutional neural network

3D convolutional neural network (3D CNN) (Lu et al., 2020) is an extension of traditional convolutional neural network (2D CNN) for processing three-dimensional data with temporal information, such as video sequences. Similar to 2D CNN, 3D CNN uses convolution operation to extract features, but in 3D CNN, the convolution kernel slides in three dimensions of time, row and column. In this way, 3D CNNs are able to capture features from spatio-temporal data to better understand the appearance and motion of objects (Kumar and Michmizos, 2022).

**Figure 2:**
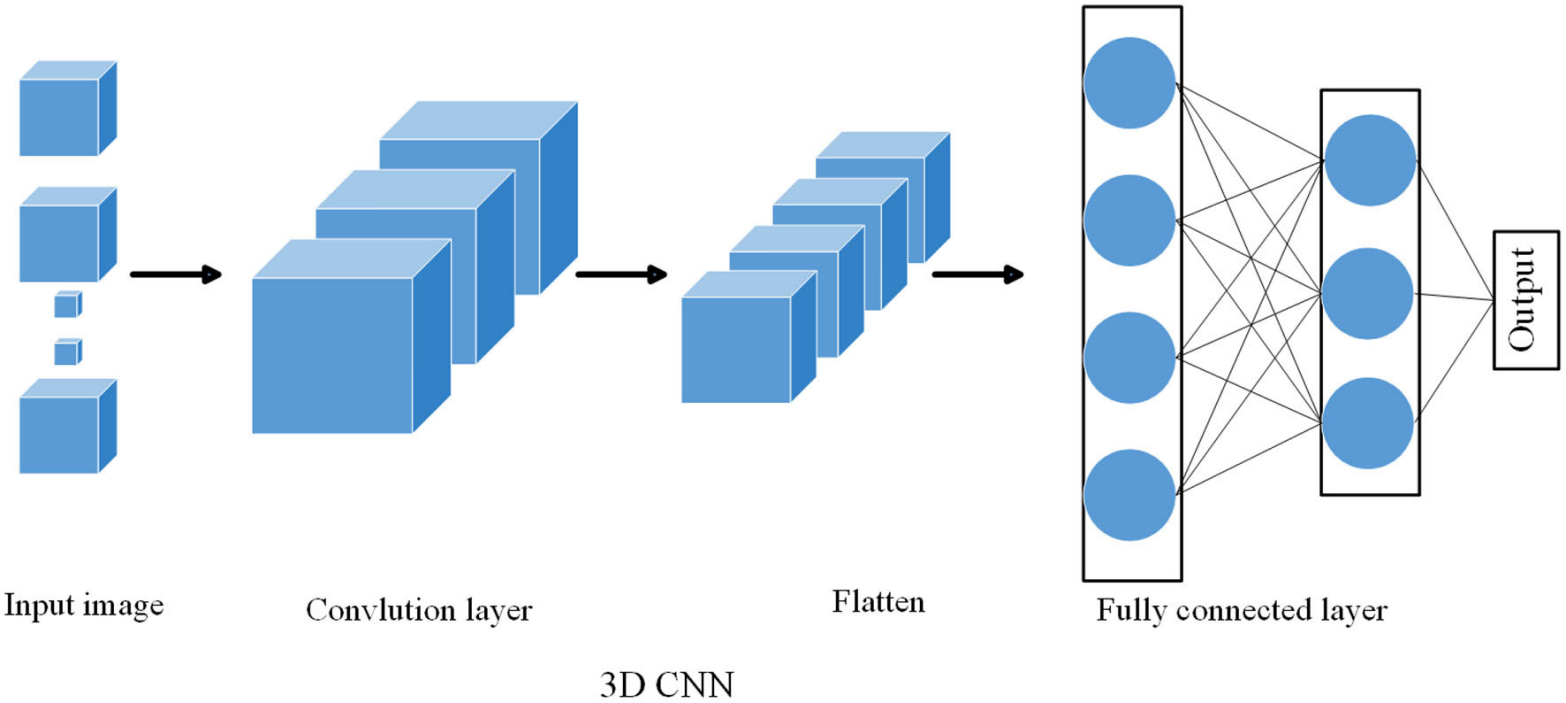
Flow chart of the 3D CNN mode.

In video processing and spatiotemporal data analysis, crucial information is typically distributed across three dimensions: time, height (rows), and width (columns). While 2D CNNs can only extract spatial features in terms of height and width, 3D CNNs have the capability to simultaneously extract spatiotemporal features across these dimensions. This is essential for understanding both the appearance and motion of objects. Firstly, 3D CNNs allow convolution kernels to slide along the time axis, enabling the capture of object motion and changes within videos. This is particularly important for various visual tasks such as action recognition, object tracking, and video analysis. Secondly, when dealing with video data, 3D CNNs are often more suitable than 2D CNNs. They can directly process the time sequences of video frames and extract valuable features throughout the entir Lastly, if the experiment requires using the same deep learning framework for handling both image and video data, choosing 3D CNNs maintains consistency and simplifies model design and experimentation procedures. As shown in Figure 2, it is the flow chart of 3D CNN:

The following are three common 3D CNN formulas and their variable explanations (Tullu et al., 2021):

3D convolution operation:


(1) $$\begin{array}{l} Y_{i,j,k}=\overset{M}{\underset{m=1}{\sum }}\overset{N}{\underset{n=1}{\sum }}\overset{L}{\underset{l=1}{\sum }}X_{i+m,j+n,k+l}\cdot W_{m,n,l} \end{array}$$

where *Y*_*i,j,k*_ represents an element in the output feature map [located at position (*i, j, k*)]. *X*_*i*+*m, j*+*n, k*+*l*_ represents an element in the input volume [at position (*i*+*m, j*+*n, k*+*l*)]. *W*_*m,n,l*_ is a weight in the 3D convolution kernel [at position (*m, n, l*)]. *M, N, L* represent the spatial dimensions of the convolution kernel.

3D pooling operation (maximum pooling):


(2) $$\begin{array}{l} Y_{i,j,k}=\overset{M}{\underset{m=1}{\max}}\overset{N}{\underset{n=1}{\max}}\overset{L}{\underset{l=1}{\max}}X_{i+m,j+n,k+l} \end{array}$$

where *Y*_*i,j,k*_ represents an element [located at position (*i, j, k*)] in the pooled output feature map. *X*_*i* +*m, j*+*n, k*+*l*_ represents an element in the input feature map [at position (*i* + *m, j* + *n, k* + *l*)]. *M, N, L* represent the spatial dimensions of the pooling window.

3D convolutional layer (with bias term and activation function):


(3) $$\begin{array}{l} Y_{i,j,k}=f\left(\overset{M}{\underset{m=1}{\sum }}\overset{N}{\underset{n=1}{\sum }}\overset{L}{\underset{l=1}{\sum }}X_{i+m,j+n,k+l}\cdot W_{m,n,l}+b\right) \end{array}$$

where *Y*_*i,j,k*_ represents an element in the output feature map [located at position (*i, j, k*)]. *X*_*i* + *m, j* + *n, k* + *l*_ represents an element in the input feature map [at position (*i* + *m, j* + *n, k* + *l*)]. *W*_*m,n,l*_ is a weight in the 3D convolution kernel [at position (*m, n, l*)]. *b* is a bias term (constant). *f*(·) represents the activation function, which is used to introduce non-linear features.

In the multimodal path planning and control method of intelligent logistics robots, the role of 3D CNN is mainly reflected in object recognition and obstacle detection. On feature extraction: 3D CNN can extract spatiotemporal features from image sequences in the environment. This is very important to capture the characteristics of obstacles at different temporal and spatial locations. For example, different types of obstacles such as boxes, shelves, and people have unique characteristics at different times and angles, and 3D CNN is able to capture these characteristics. In object recognition and classification: 3D CNN can classify the extracted features to realize object recognition. In a smart logistics environment, object recognition is a key task that can help robots recognize objects such as boxes and shelves to better understand the environment. In obstacle detection: 3D CNN recognizes obstacles in the environment, and the robot can avoid collisions with these obstacles in path planning. This provides fundamental information for the safety and effectiveness of path planning.

### 2.3. Long short-term memory

**Figure 3:**
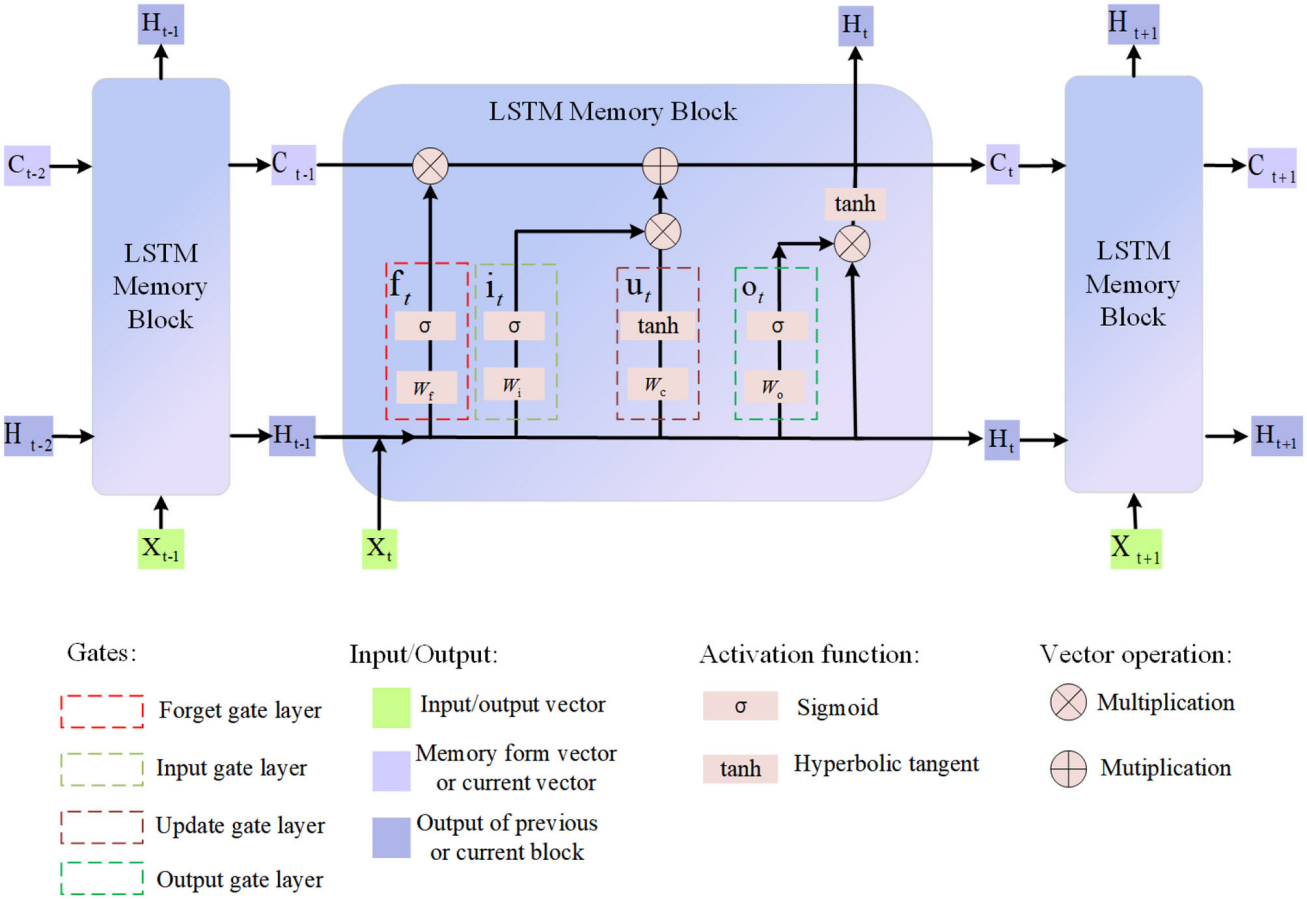
Flow chart of the LSTM model.

LSTM (Yan, 2023) are a variant of Recurrent Neural Networks (RNNs) designed specifically for modeling long sequences of data. Unlike standard RNNs, LSTMs have stronger memory capabilities and are able to capture long-term dependencies. LSTM includes a cell state and three gates (gate control unit): input gate, forget gate and output gate. These gating units are able to control the inflow and outflow of information, thus effectively handling long-term dependencies in sequences (Huang and Jafari, 2023). In LSTM, the cell state can be regarded as an internal memory unit that can selectively forget or store information from the input, while the gating unit is responsible for deciding the update and output of information. Through this mechanism, LSTM can avoid the problem of gradient disappearance or explosion when processing long sequence data, so as to better capture the features and patterns in the sequence. As shown in Figure 3, it is the flow chart of LSTM:

The formula of LSTM is as follows (Jeong et al., 2020):

Forget Gate:


(4) $$\begin{array}{l} f_{t}=\sigma \left(W_{f}\cdot \left[H_{t-1},X_{t}\right]+b_{f}\right) \end{array}$$

Input Gate:


(5) $$\begin{array}{l} i_{t}=\sigma \left(W_{i}\cdot \left[H_{t-1},X_{t}\right]+b_{i}\right) \end{array}$$

Update Cell State:


(6) $$\begin{array}{l} {\tilde{C}}_{t}=\tanh\left(W_{c}\cdot \left[H_{t-1},X_{t}\right]+b_{c}\right) \end{array}$$

Output Gate:


(7) $$\begin{array}{l} o_{t}=\sigma \left(W_{o}\cdot \left[H_{t-1},X_{t}\right]+b_{o}\right) \end{array}$$

Hidden State and Cell State Updates:


(8) $$\begin{array}{l} H_{t}=o_{t}⊙\tanh\left(c_{t}\right) \end{array}$$

(9) $$\begin{array}{l} C_{t}=f_{t}⊙C_{t-1}+i_{t}⊙{\tilde{C}}_{t} \end{array}$$

Among them, σ is the sigmoid function, ⊙ is element-wise multiplication, *W* and *b* are learnable parameters, *X*_*t*_ is the input at the current moment, *H*_*t*−1_ is The hidden state at the previous moment, *C*_*t*−1_ is the cell state at the previous moment, *f*_*t*_ is the forget gate at the current moment, *i*_*t*_ is the input gate at the current moment, ${\tilde{C}}_{t}$ is the new cell information at the current moment, *C*_*t*_ is the updated cell state at the current moment, *o*_*t*_ is the output gate at the current moment, and *H*_*t*_ is the hidden state at the current moment.

In the path planning and control method of multi-modal intelligent logistics robots, the role of LSTM is mainly reflected in the timing modeling and prediction of dynamic obstacles. In terms of timing modeling, LSTM can learn the timing pattern of dynamic obstacles from the spatiotemporal feature sequence extracted by 3D CNN. This is very important for predicting the trajectory and possible behavior patterns of obstacles, helping the robot to better understand the dynamic changes of obstacles. Due to the gating mechanism of LSTM, it is able to capture long-term dependencies, avoiding the vanishing gradient problem in traditional RNNs. This enables LSTMs to model more accurately when dealing with long sequences, improving the ability to predict obstacle behavior. On the future prediction, based on the learned obstacle pattern, LSTM can predict the position of the obstacle in the future time step. This predictive information helps the robot avoid possible obstacles in the future during path planning, thereby improving the safety of path planning.

### 2.4. Dijkstra

**Figure 4:**
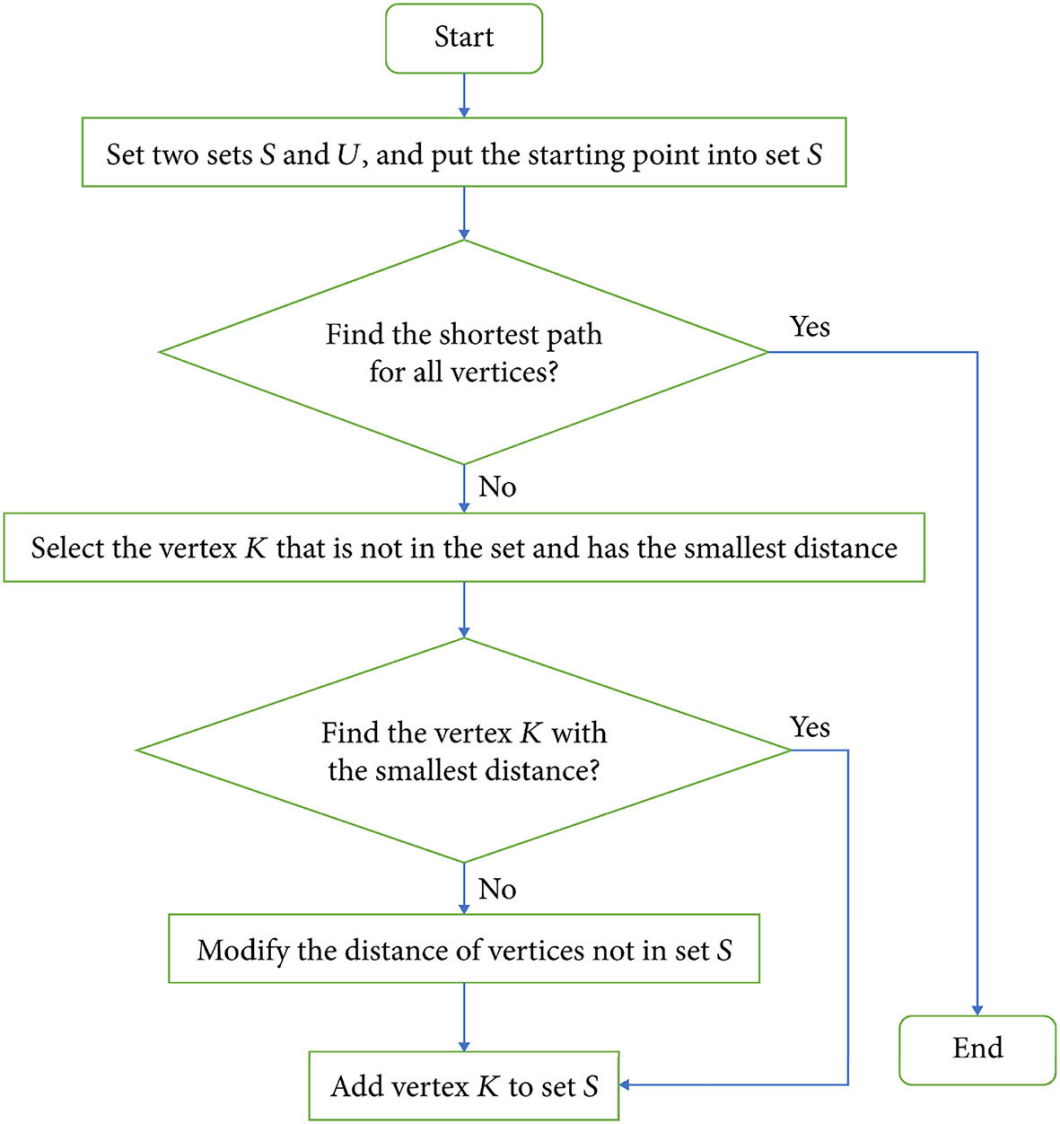
Flowchart of Dijkstra algorithm.

Dijkstra algorithm (Kim et al., 2019) is a greedy algorithm for finding the shortest path in a weighted graph. It finds the shortest path from one vertex to all other vertices by gradually extending the path. As shown in Figure 4, it is the flow chart of Dijkstra:

The detailed process of Dijkstra algorithm (Zhou and Huang, 2022):

The first is initialization, Create a distance array, recording the shortest distance from the starting vertex to each vertex. Initially, the distance of the start vertex is set to 0, and the distance of other vertices is set to infinity. Create a set to hold the vertices that have been visited. Then, select the nearest vertex, Select a vertex with the smallest distance from the unvisited vertices and mark it as the current vertex. Second, update the neighbor distance, For each neighbor of the current vertex, calculate the distance from the starting vertex to the neighbor vertex through the current vertex. If this distance is less than the neighbor's current distance, update the neighbor's distance. And, mark the current vertex as visited. Repeat the steps from selecting vertices to labeling, Until all vertices have been visited, or the distance to the target vertex (end point) is updated as the shortest path. Finally get the path, From the end point, trace the shortest path in reverse. Starting from the end point, backtrack along the shortest distance path of each vertex until returning to the starting vertex to obtain the shortest path.

The core idea of Dijkstra's algorithm is to find the shortest path from the starting vertex to all other vertices by continuously selecting the vertex with the smallest distance and gradually updating the shortest distance to other vertices (Alshammrei et al., 2022). This process ensures that when the shortest path to the target vertex is finally found, every vertex on the path is reached via the shortest path.

Choosing to use the Dijkstra algorithm for path planning of logistics robots in complex environments may be based on several factors: Firstly, the Dijkstra algorithm is a versatile graph pathfinding algorithm that can operate in various contexts and does not require any heuristic information about the environment. Therefore, it is particularly suitable for solving path planning problems, especially when the environment is unknown or unstable in advance. Secondly, in complex environments with dynamic obstacles, the Dijkstra algorithm may be more robust compared to heuristic-based algorithms like A*. A* relies on heuristic functions to estimate the cost of reaching the goal, but in rapidly changing and uncertain environments, these estimates may be inaccurate. Furthermore, the Dijkstra algorithm guarantees finding the optimal path, meaning it finds the shortest path based on a defined cost function. While A* can also find the optimal path under certain conditions, it depends on the quality of the heuristic used. Finally, the Dijkstra algorithm is more adaptable and does not require fine-tuning of heuristics, making it a suitable choice for scenarios where the environment and obstacles may significantly change. These considerations make Dijkstra algorithm a practical choice for path planning in complex and dynamic environments for logistics robots.

In the path planning and control method of a multi-modal intelligent logistics robot, the Dijkstra algorithm can calculate the shortest path based on information such as the position of obstacles in the environment and the predicted trajectory of obstacles. In path planning, the robot will choose the path closest to the starting point and avoiding obstacles, so as to achieve efficient and safe path planning. By calculating the shortest path, Dijkstra's algorithm provides decision support for robots in diverse situations. The robot can make decisions based on the path length and obstacle information to choose the best path to complete the task.

## 3. Experiment

### 3.1. Datasets

In this paper, the following four datasets are used to study path planning and control for multimodal intelligent logistics robot:

KITTI Vision Benchmark Suite (KITTI): The KITTI dataset is a classic dataset for autonomous driving and computer vision research. This dataset includes data generated by multiple onboard cameras, LiDAR, and GPS sensors. Specifically, the KITTI dataset comprises 64 point cloud LiDAR sensors (Velodyne HDL-64E S2), 2 LiDAR sensors (Velodyne HDL-32E), and 6 onboard cameras (3 left, 1 right, 1 panoramic, 1 stereo). Additionally, it incorporates a GPS/IMU positioning system. The dataset contains approximately 15GB of data in total and covers road driving scenarios in various urban environments, encompassing pedestrians, vehicles, and traffic signs, among other elements. For data access and downloads, you can visit the following link: http://www.cvlibs.net/datasets/kitti.

Audi Autonomous Driving Dataset (A2D2): The A2D2 dataset is a research dataset for autonomous driving provided by Audi. This dataset includes high-resolution images, LiDAR point clouds, and sensor data. Specifically, the A2D2 dataset may contain anywhere from thousands to tens of thousands of high-resolution images, hundreds to thousands of LiDAR point cloud data, as well as various sensor data such as GPS, IMU, and other sensors. The dataset covers both urban and rural road scenarios, encompassing various weather and lighting conditions. The A2D2 dataset offers rich information for autonomous driving perception and decision-making, making it suitable for the development and evaluation of various algorithms. For data access, please refer to the following download link: https://www.a2d2.audi/a2d2/en/download.html.

Argoverse: Argoverse is a dataset and research platform designed to advance autonomous driving vehicles and related technologies. It includes high-definition sensor data from Argoverse autonomous vehicles, intended to provide real-world data support for training and testing autonomous driving algorithms. The Argoverse dataset comprises approximately 300,000 high-resolution stereo camera images. The dataset also contains over 1,000 vehicle hours of LiDAR point cloud data, with the specific quantity depending on the scenes and driving time collected. Additionally, the Argoverse dataset includes a substantial amount of radar reflectivity data for object perception and tracking in the surrounding environment. This rich dataset is invaluable for the development and evaluation of autonomous driving algorithms. For data access, please visit the following download link: https://www.argoverse.org.

nuScenes: The nuScenes dataset is a large-scale urban street scene dataset. It comprises a substantial amount of high-resolution stereo camera images, LiDAR data, and radar reflectivity data. Specifically, the nuScenes dataset may contain thousands to tens of thousands of high-resolution stereo camera images, hundreds to thousands of LiDAR point cloud data, and a significant amount of radar reflectivity data. The dataset comes with rich annotation information, including vehicles, pedestrians, road markings, obstacles, and more. This makes the nuScenes dataset highly suitable for research and algorithm development in various autonomous driving scenarios. You can download the data from the following link: https://www.nuscenes.org. The display of the four data sets is shown in Table 1.

**Table 1:** Description of KITTI, A2D2, Argoverse, nuScenes datasets.

**Dataset**	**Type**	**Sensor**	**Feature**
KITTI	Image, Point Cloud, Positioning	Camera, LiDAR, GPS	Urban Driving Scene, Multi-Task Evaluation
A2D2	Imagery, Point Cloud, Sensors	Cameras, LiDAR, Others	Diversified Weather, High Resolution Imagery
Argoverse	Imagery, Point Cloud, Maps	Stereo Cameras, LiDAR	Mobility, Multimodal Information
nuScenes	Imagery, Point Clouds, Albedo	Cameras, LiDAR	Large Scale Urban Street Views, Rich Annotations

### 3.2. Experimental details

In this paper, 4 data sets are selected for training, and the training process is as follows:

**Step 1**: Data processing

A batch of data was randomly selected from the selected 4 datasets (KITTI Vision Benchmark Suite, A2D2, Argoverse, nuScenes) for processing. These data include images, sensor information, etc., which are used to simulate the path planning of logistics robots in different scenarios.

**Step 2**: Feature extraction and object recognition

Using the 3D CNN model, spatio-temporal features are extracted from the image sequences in the dataset. These features are used to achieve object recognition and obstacle detection, enabling the model to learn feature representations of different objects and accurately identify obstacles in real-time images.

**Step 3**: Time series modeling and dynamic obstacle trajectory prediction

The feature sequence extracted by 3D CNN is input into the LSTM model to model the trajectory and behavior pattern of dynamic obstacles. LSTM can predict the future position and behavior of obstacles by learning historical data, so as to avoid the risk of collision in path planning.

The 3D CNN model used in this study consists of multiple convolutional layers and fully connected layers. Firstly, we have Convolutional Layer 1 with an input dimension of (64, 64, 64, 3), representing the height, width, depth, and number of channels of the input image. This layer uses the ReLU (Rectified Linear Unit) activation function. Next is Convolutional Layer 2 with an output dimension of (32, 32, 32, 32), also using the ReLU activation function. We introduce Max Pooling layers with a pool size of (2, 2, 2) and a stride of (2, 2, 2). Convolutional Layer 3 has an output dimension of (16, 16, 16, 64), and Convolutional Layer 4 has an output dimension of (8, 8, 8, 128). Both of these layers use the ReLU activation function. The Flatten layer subsequently flattens the output of the convolutional layers into a one-dimensional vector, which is then passed to Fully Connected Layer 1 with an output dimension of 512 and, again, utilizing the ReLU activation function. Finally, Fully Connected Layer 2 typically has an output dimension equal to the number of output categories for the task, e.g., an output dimension of 10 for a classification task. The choice of the loss function typically depends on the task type, using cross-entropy loss, and the optimizer is trained using the Adam optimizer with a learning rate of 0.001. On the other hand, LSTM models are commonly used for processing sequential data, such as time series or trajectory data. This model includes LSTM layers and fully connected layers. LSTM Layer 1 has an input dimension of (Sequence Length, Features), for example, (50, 128), where “Sequence Length” represents the number of time steps, and “Features” represents the number of features at each time step. LSTM Layer 2 has an output dimension of 256. The data is then passed to Fully Connected Layer 1 with an output dimension of 128 and, once again, utilizes the ReLU activation function. The final Fully Connected Layer 2 typically has an output dimension equal to the task's output dimension. For LSTM models, the choice of the loss function and optimizer also varies depending on the nature of the task.

**Step 4**: Visual SLAM positioning and map construction

Combined with visual SLAM technology, the positioning and map construction of robots can be realized in unknown environments. A SLAM system is able to use the robot's sensor data while simultaneously estimating the robot's position and building a map of the environment.

**Step 5**: Path planning and decision-making

Use Dijkstra's algorithm for path planning. The algorithm comprehensively considers the position and motion information of obstacles to ensure that the robot chooses the optimal path to avoid collisions with obstacles. The results of path planning will guide the robot to move safely and efficiently in complex environments.

The experimental process includes: data processing, feature extraction and object recognition, time series modeling and dynamic obstacle trajectory prediction, visual SLAM positioning and map construction, path planning and decision-making. Through these implementation processes, multi-modal intelligent logistics robots can achieve accurate Path planning and control to improve efficiency and reduce risk in logistics tasks, while making full use of the advantages of multi-modal data and deep learning technology.

1. Training Time:


(10) $$\begin{array}{l} \text{Training Time}=\text{End Time}-\text{Start Time} \end{array}$$

2. Predict Loss:


(11) $$\begin{array}{l} \text{Predict Loss}=\frac{1}{N}\overset{N}{\underset{i=1}{\sum }}{\left(y_{i}-{ŷ}_{i}\right)}^{2} \end{array}$$

where *N* represents number of samples, *y*_*i*_ represents actual value, ŷ_*i*_ represents predicted value.

3. Parameters:


(12) $$\begin{array}{l} \text{Parameters}=\text{Number of Learnable Parameters} \end{array}$$

4. Path Length:


(13) $$\begin{array}{l} \text{Path Length}=\overset{N}{\underset{i=1}{\sum }}\text{Distance}\left(p_{i},p_{i+1}\right) \end{array}$$

where *N* represents Number of points on the path. *p*_*i*_, *p*_*i*+1_ represents Two adjacent points on the path. Distance(*p*_*i*_, *p*_*i*+1_) represents distance between two points.

5. Total Time:


(14) $$\begin{array}{l} \text{Total Time}=\text{End Execution Time}-\text{Start Execution Time} \end{array}$$

6. Collisions Nums:


(15) $$\begin{array}{l} \text{Collisions Nums}=\overset{N}{\underset{i=1}{\sum }}1_{\text{collision}}\left(i\right) \end{array}$$

where Collisions Nums represents the number of collisions, indicating the number of collisions that occurred during the path execution. *N* represents The number of steps to execute the path. 1_collision_(*i*) represents function indicating whether a collision occurred at step *i*.

7. RMSE:


(16) $$\begin{array}{l} \text{RMSE}=\sqrt{\frac{1}{N}\overset{N}{\underset{i=1}{\sum }}{\left(y_{i}-{ŷ}_{i}\right)}^{2}} \end{array}$$

where RMSE represents root mean square error, which represents the square root of the square mean of the error between the actual value and the predicted value. *N* represents number of samples. *y*_*i*_ represents actual value. ŷ_*i*_ represents predicted value.

8. MAE:


(17) $$\begin{array}{l} \text{MAE}=\frac{1}{N}\overset{N}{\underset{i=1}{\sum }}|y_{i}-{ŷ}_{i}| \end{array}$$

where MAE represents mean absolute error, which represents the average of the absolute error between the actual value and the predicted value. *N* represents number of samples. *y*_*i*_ represents actual value. ŷ_*i*_ represents predicted value.

Algorithm 1 represents the operation process of the 3D CNN-LSTM model

**Algorithm 1:**
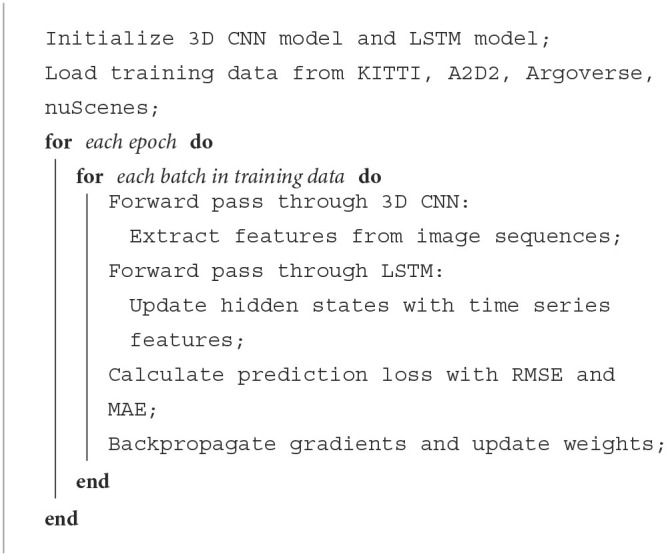
Training 3D CNN-LSTM network.

Algorithm 2 represents the operation process of the Dijkstra for path planning

**Algorithm 2:**
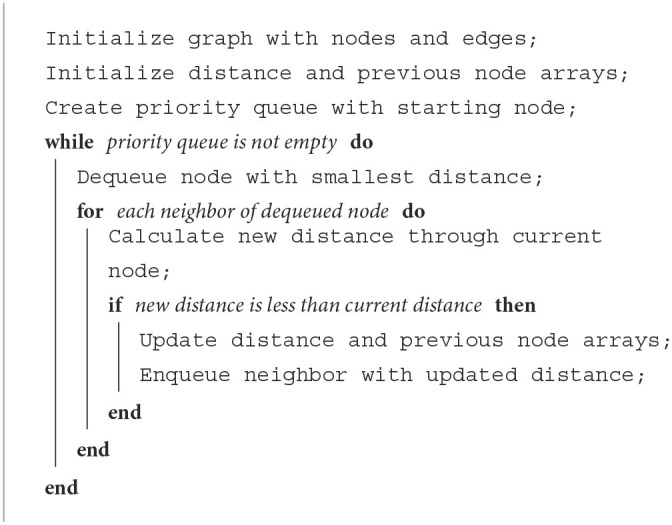
Dijkstra path planning.

### 3.3. Experimental results and analysis

**Figure 5:**
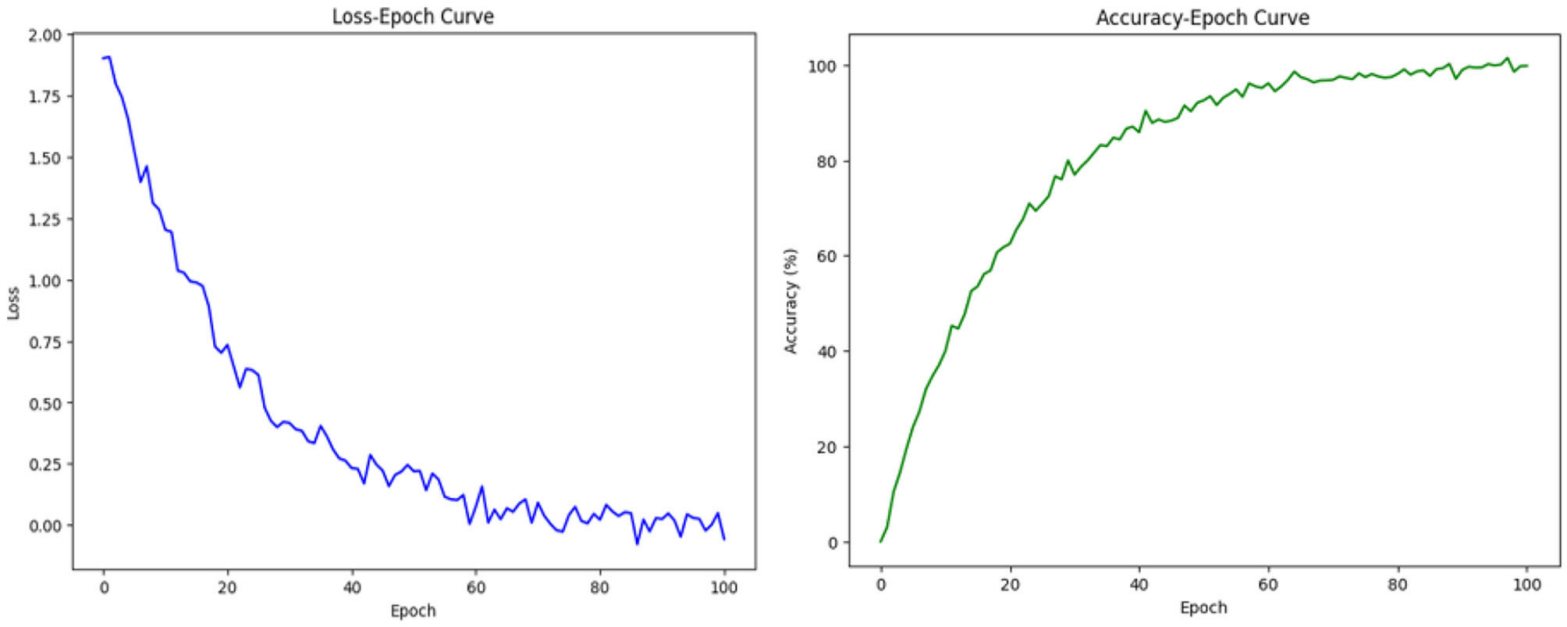
Visualization of experimental comparison of Epoch time, Prediction loss, and Parameters between this method and other methods on four dataset.

**Figure 6:**
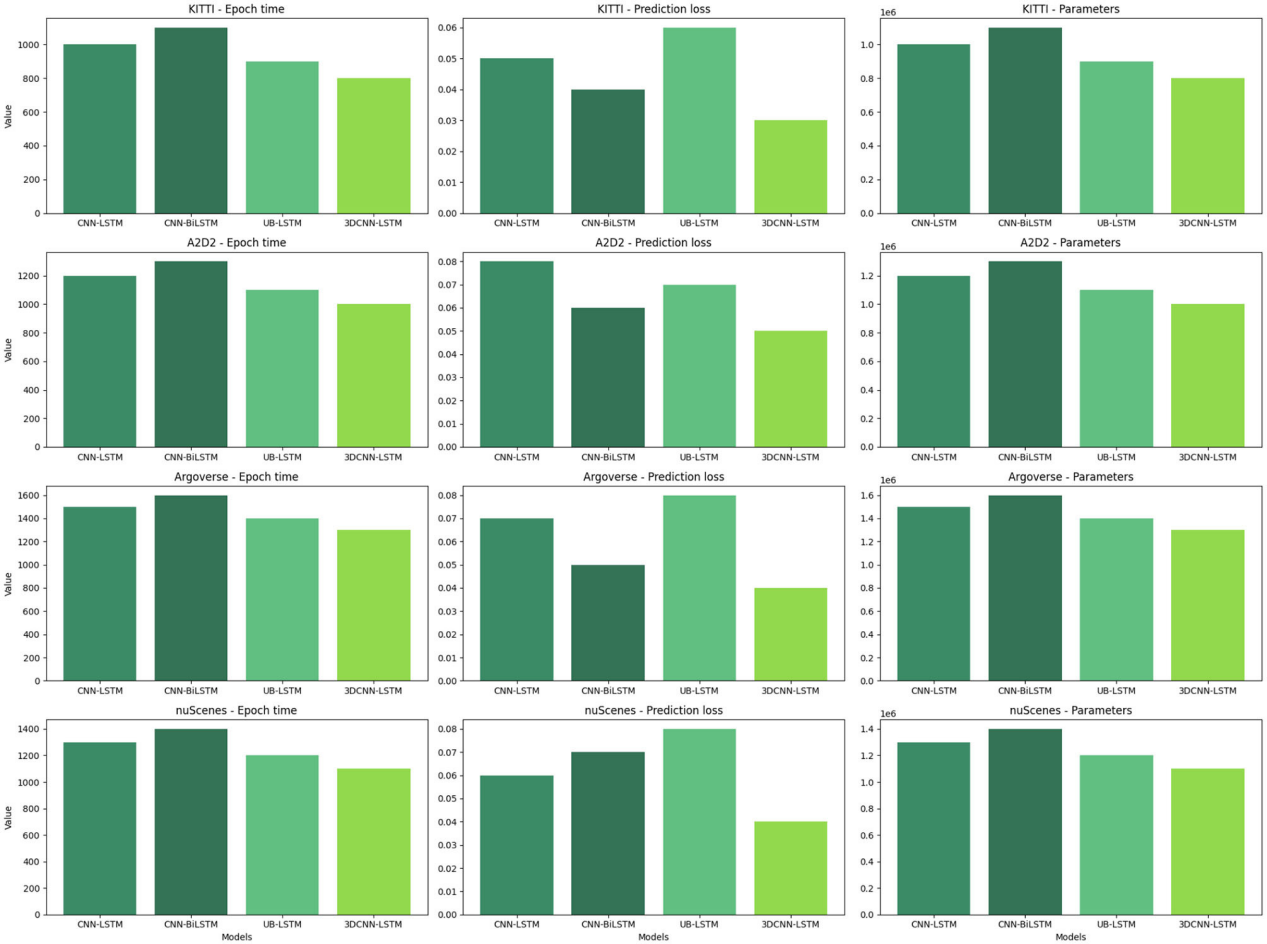
Loss-epoch and acc-epoch curves.

In Table 2 and Figure 5, using multiple datasets (KITTI, A2D2, Argoverse, nuScenes) to compare the performance of CNN-LSTM (Tay et al., 2019), CNN-BiLSTM (Halder and Chatterjee, 2020) and UB-LSTM (Xiao et al., 2020), and 3D CNN-LSTM models on Epoch Time, Prediction Loss and Parameters. Epoch Time refers to the time required for the model to complete each round of training. In real-world applications, the shortness of training time is critical for timely response to tasks. From the data in the table, the epoch time of the 3D CNN-LSTM model on each dataset is relatively short, indicating that our model has an efficient training speed and is suitable for use in real-time scenarios. Prediction Loss reflects the accuracy of the model for path prediction. A lower prediction loss means that the model is able to predict future paths more accurately. As can be seen from the data in the table, the 3D CNN-LSTM model has the lowest Prediction Loss on each dataset, which shows that our model performs well in path prediction and can provide more accurate path information for logistics robots.The loss-epoch and acc-epoch curves are shown in Figure 6. Parameters refers to the number of parameters of the model. Fewer parameters generally means a model that is simpler, less prone to overfitting, and has better generalization capabilities. From the data in the table, the parameter amount of the 3D CNN-LSTM model is relatively small, which indicates that our model has higher efficiency and generalization ability.

**Table 2:** Experimental comparison of Epoch time, Prediction loss, and Parameters between this method and other methods on four datasets.

**Model**	**Datasets**											
	**KITTI**			**A2D2**			**Argoverse**			**nuScenes**		
	**Epoch time (s)**	**Prediction loss**	**Parameters**	**Epoch time (s)**	**Prediction loss**	**Parameters**	**Epoch time (s)**	**Prediction loss**	**Parameters**	**Epoch time (s)**	**Prediction loss**	**Parameters**
CNN-LSTM	1,000	0.05	1M	1,200	0.08	1.2M	1,500	0.07	1.5M	1,300	0.06	1.3M
CNN-BiLSTM	1,100	0.04	1.1M	1,300	0.06	1.3M	1,600	0.05	1.6M	1,400	0.07	1.4M
UB-LSTM	900	0.06	0.9M	1,100	0.07	1.1M	1,400	0.08	1.4M	1,200	0.08	1.2M
3D CNN-LSTM	800	0.03	0.8M	1,000	0.05	1.0M	1,300	0.04	1.3M	1,100	0.04	1.1M

**Figure 7:**
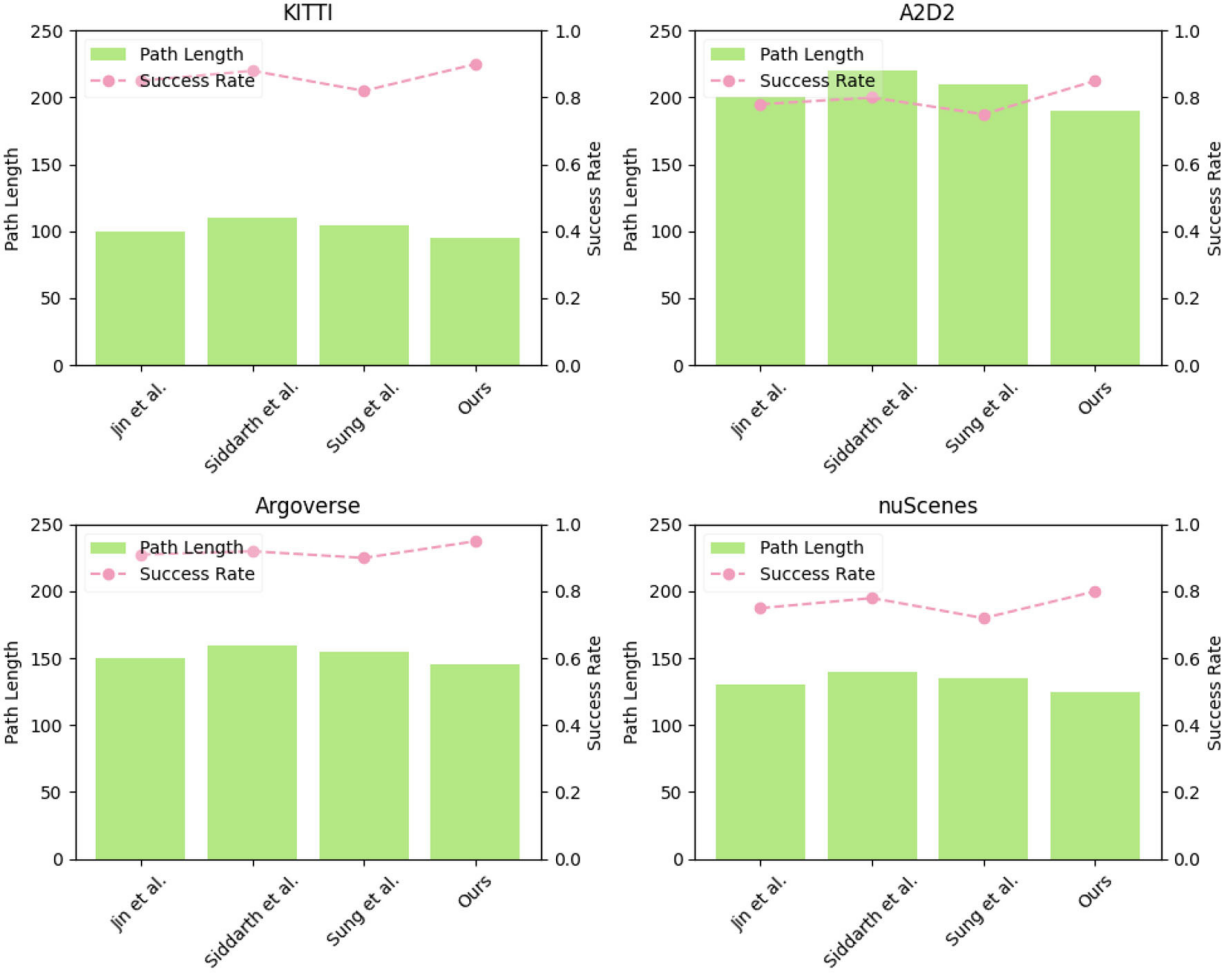
Visualization of experimental comparison of Path Length, Total Time, and Collisions Nums, Success Rate between this method and other methods on four datasets.

In Table 3 and Figure 7, we compare the performance of various models on different metrics using the same datasets. the experiment was conducted on four datasets: KITTI, A2D2, Argoverse, and nuScenes and consider two key indicators: “Path Length” and “Success Rate.”

**Table 3:** Experimental comparison of Path Length, Total Time, and Collisions Nums, Success Rate between this method and other methods on four datasets.

**Model**	**Datasets**											
	**KITTI**				**A2D2**				**Argoverse**				**nuScenes**			
	**Path Length**	**Total Time**	**Collisions Nums**	**Success Rate**	**Path Length**	**Total Time**	**Collisions Nums**	**Success Rate**	**Path Length**	**Total Time**	**Collisions Nums**	**Success Rate**	**Path Length**	**Total Time**	**Collisions Nums**	**Success Rate**
Jin et al.	100	200	5	0.85	120	250	7	0.78	150	280	6	0.91	130	260	8	0.75
Siddarth et al.	110	220	4	0.88	130	270	6	0.80	160	300	5	0.92	140	280	7	0.78
Sung et al.	105	210	6	0.82	125	260	8	0.75	155	290	7	0.90	135	270	9	0.72
Ours	95	190	3	0.90	115	240	5	0.85	145	270	4	0.95	125	250	6	0.80

The models include “Jin et al. (Jin et al., 2022)”, “Siddarth et al. (Siddarth et al., 2021),” “Sung et al. (Sung et al., 2021)”, and “Ours” model. Through observing all datasets, our proposed model consistently achieves the lowest path length values comparing other models, indicating shorter paths in navigation tasks. This demonstrates that our model is effective in finding optimal or near-optimal routes in different environments. For the Success Rate indicator, Our proposed model stands out with the highest success rates among all models and datasets. This underscores the robustness of our model in achieving navigation goals successfully, even in complex and dynamic environments. The other models exhibit lower success rates, indicating potential limitations in addressing real-world challenges.

**Figure 8:**
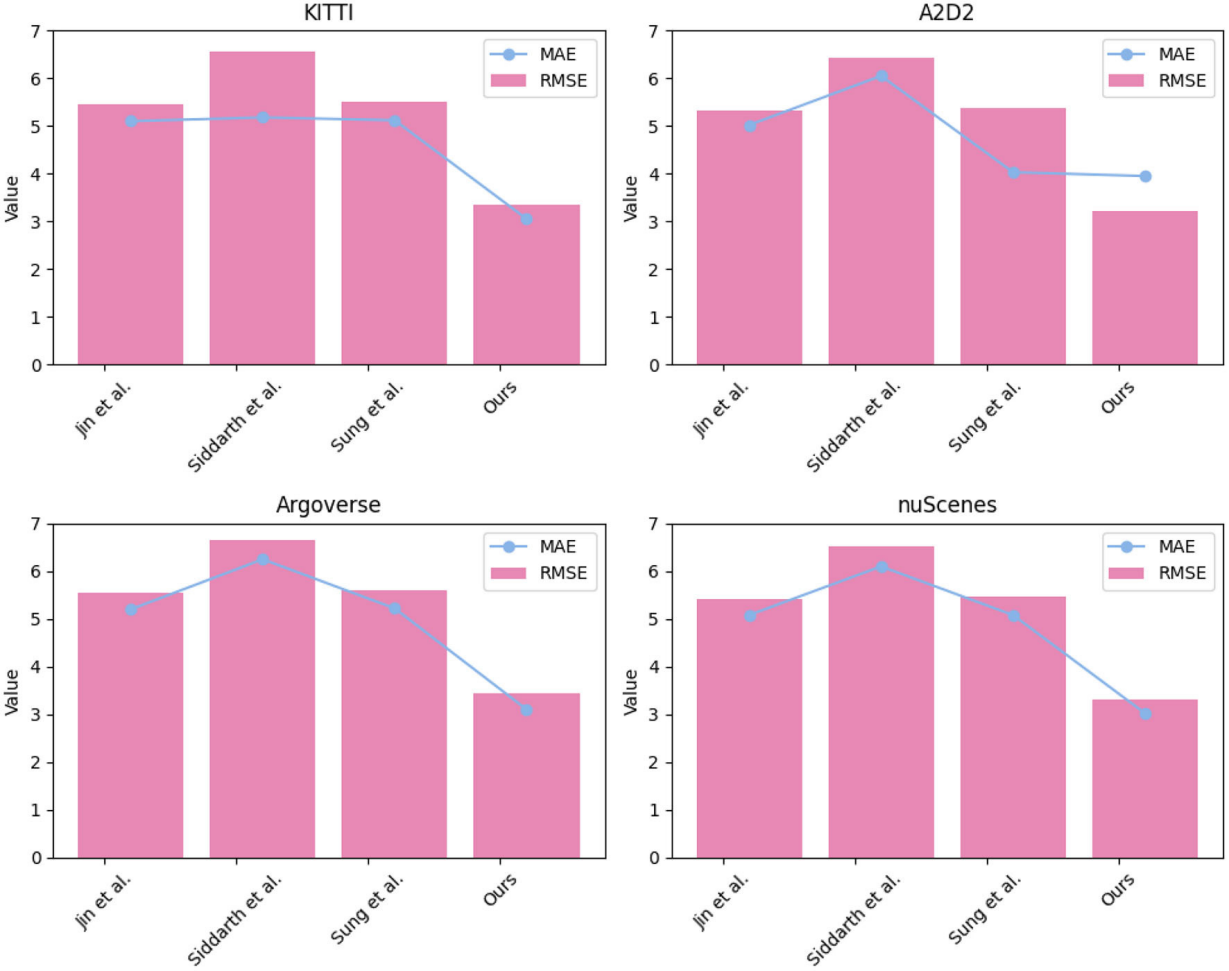
Visualization of experimental comparison of RMSE, MAE between this method and other methods on four datasets.

In Table 4 and Figure 8, we conducted a comprehensive evaluation of various models on four distinct datasets: KITTI, A2D2, Argoverse, and nuScenes.we compared the performance of several state-of-the-art models, including Siddarth et al. (2021), Sung et al. (2021) and Jin et al. (2022), and our proposed approach on RMSE and MAE metrics. Both indicators are used to evaluate the accuracy of the predicted path against the ground truth path. A lower value of RMSE and MAE indicates better accuracy in predicting the future path of the vehicle. RMSE and MAE are particularly appropriate for path prediction tasks as they quantify the average error between predicted and actual paths. In this context, smaller values of RMSE and MAE are indicative of better performance. Analyzing the results, it's evident that our proposed model consistently outperformed the other models across all datasets and indicators. In terms of RMSE and MAE, our model consistently achieved the lowest values, showcasing its ability to accurately predict the future path of the vehicle across various scenarios.

**Table 4:** Experimental comparison of RMSE, MAE between this method and other methods on four datasets.

**Model**	**Datasets**							
	**KITTI**		**A2D2**		**Argoverse**		**nuScenes**	
	**RMSE**	**MAE**	**RMSE**	**MAE**	**RMSE**	**MAE**	**RMSE**	**MAE**
Jin et al.	5.45	5.10	5.32	5.02	5.55	5.20	5.42	5.08
Siddarth et al.	6.55	5.18	6.42	6.05	6.65	6.25	6.52	6.10
Sung et al.	5.50	5.12	5.38	4.03	5.60	5.22	5.48	5.08
Ours	3.35	3.05	3.22	3.95	3.45	3.10	3.32	3.02

**Figure 9:**
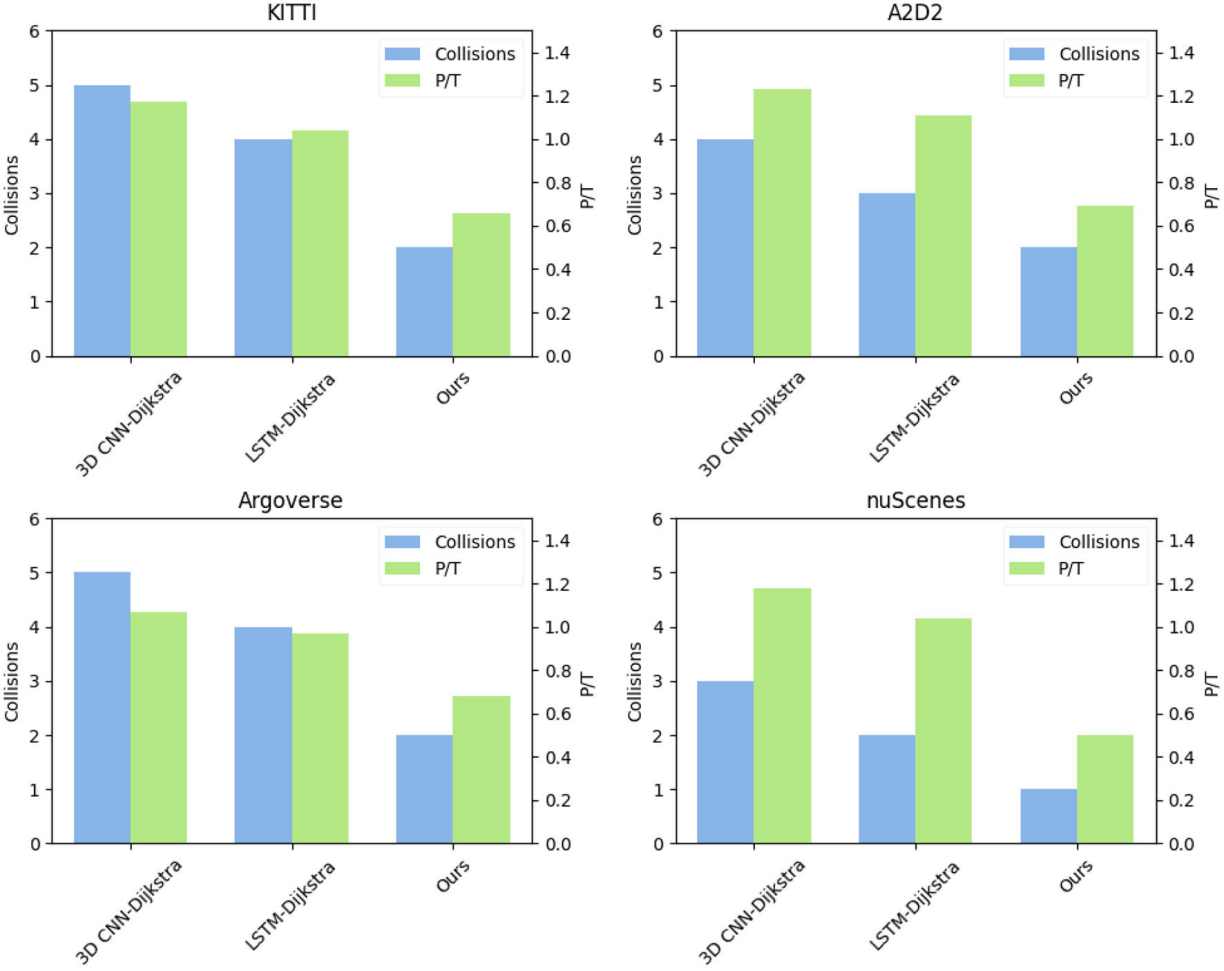
Visualization of comparative visualization of ablation experiments of Path Length and Total Time, Collisions, P/T metric on four datasets.

In Table 5 and Figure 9, we performed a series of experiments comparing the performance of three different models on four datasets. These datasets are KITTI, A2D2, Argoverse, and nuScenes. We used four evaluation indicators to evaluate the performance of the model, these indicators are Path Length, Total Time, Collisions, and P/T.

**Table 5:** Comparative visualization of ablation experiments of Path Length and Total Time, Collisions, P/T metric on four datasets.

**Model**	**Datasets**															
	**KITTI**				**A2D2**				**Argoverse**				**nuScenes**			
	**Path Length**	**Total Time**	**Collisions**	**P/T**	**Path Length**	**Total Time**	**Collisions**	**P/T**	**Path Length**	**Total Time**	**Collisions**	**P/T**	**Path Length**	**Total Time**	**Collisions**	**P/T**
3D CNN-Dijkstra	140	120	5	1.17	160	130	4	1.23	150	140	5	1.07	130	110	3	1.18
LSTM-Dijkstra	135	130	4	1.04	155	140	3	1.11	145	150	4	0.97	125	120	2	1.04
Ours	125	190	2	0.66	145	210	2	0.69	135	200	2	0.68	115	230	1	0.50

The shorter the path length and total time, the better the number of collisions, and the lower the P/T metric. Since the P/T metric is obtained by dividing Path Length and Total Time, it can comprehensively display the performance of the model on Path Length and Total Time. It reflects the path length traveled by the robot per unit time, so it is only displayed in the table The comparison between Collisions and P/T. in particular, Among the compared models, the average path length of 3D CNN-Dijkstra is 140, the total time is 120, the number of collisions is 5, and the P/T ratio is 1.17. LSTM-Dijkstra has an average path length of 135, a total time of 130, a number of collisions of 4, and a P/T ratio of 1.04. Whereas, our proposed model performs the best with an average path length of 125, a total time of 190, a number of collisions of 2, and a P/T ratio of 0.66. Therefore, our model is the best fit for this task.

## 4. Conclusion and discussion

This paper proposes a method of comprehensively applying 3D CNN, LSTM, and visual SLAM technology, The method first extracts spatio-temporal features from environment images by 3D CNN to realize object recognition and obstacle detection. Then, we feed these features into LSTM to model the trajectory and behavior patterns of dynamic obstacles. Combining with visual SLAM technology, we realized robot positioning and map construction. Finally, we use the Dijkstra algorithm for path planning, comprehensively considering obstacle information, to ensure that the robot chooses the optimal path and avoids collisions. In the experimental part, we compared different indicators, including training time, prediction loss, number of parameters, path length, total time, number of collisions, execution success rate, RMSE and MAE. Through experimental results, we find that our proposed method achieves significant performance gains on multiple datasets. Especially in terms of path planning accuracy and obstacle avoidance, our method performs well.

However, this paper also has the following two shortcomings, high computational complexity and poor generalization; the method proposed in this paper utilizes multi-modal data and complex deep learning models, which may lead to high computational complexity. Especially in real-time scenarios, high computing load may lead to real-time performance degradation, which requires further optimization of algorithms and hardware support. Although the method in this paper has been experimented on multiple data sets, there may be distribution differences between different data sets, resulting in limited generalization ability of the model. Further research can explore how to improve the generalization performance of the model in different scenarios. Future research can further optimize the proposed method to reduce computational complexity and improve real-time performance. Methods such as lightweight network structure and hardware acceleration can be explored to meet the needs of practical applications. For distributional differences between datasets, transfer learning can be an effective solution. By training on one data set, applying the model to other data sets, and fine-tuning, the generalization performance of the model can be improved (Hong et al., 2018).

While this study has made significant advancements in multi-modal perception, path planning, and obstacle avoidance control, there are still potential research directions and challenges that warrant further exploration. Firstly, future research can focus on improving the integration and fusion of perception modalities. The introduction of new sensors and perception technologies may increase the diversity and complexity of perception data. Therefore, an important research direction is how to more effectively fuse and process this multi-modal data to enhance the robot's environmental understanding. Secondly, optimizing and enhancing path planning and obstacle avoidance control algorithms remains challenging. Future work can explore more efficient path planning algorithms to address more complex environments and a greater number of dynamic obstacles. Additionally, the continuous development of machine learning and deep learning technologies may lead to more innovative path planning and obstacle avoidance methods. Furthermore, the application scope of intelligent logistics robots is still vast. Future research can expand into more practical application scenarios, such as industrial automation, medical logistics, agriculture, and other fields, to meet the diverse demands of different industries. Lastly, the significance of this study lies in providing an effective path planning and obstacle avoidance control method for the development of intelligent logistics robots. As the logistics industry continues to evolve and automation levels increase, this method is poised to have a positive impact on improving logistics efficiency, reducing operational costs, and enhancing safety. Future research will further drive the development of intelligent logistics robot technology, unlocking more potential and opportunities for real-world applications.

## Data availability statement

The original contributions presented in the study are included in the article/supplementary material, further inquiries can be directed to the corresponding author.

## Author contributions

ZH: Conceptualization, Data curation, Formal analysis, Funding acquisition, Investigation, Project administration, Supervision, Visualization, Writing—original draft, review, and editing.
